# Long-Term Fasting-Induced Ketosis in 1610 Subjects: Metabolic Regulation and Safety

**DOI:** 10.3390/nu16121849

**Published:** 2024-06-13

**Authors:** Franziska Grundler, Robin Mesnage, Philip M. M. Ruppert, Demetrios Kouretas, Françoise Wilhelmi de Toledo

**Affiliations:** 1 Buchinger Wilhelmi Clinic, Wilhelmi-Beck-Straße 27, 88662 Überlingen, Germany; franziska.grundler@buchinger-wilhelmi.com (F.G.); francoise.wilhelmi@buchinger-wilhelmi.com (F.W.d.T.); 2 Gene Expression and Therapy Group, King’s College London, Faculty of Life Sciences & Medicine, Department of Medical and Molecular Genetics, 8th Floor, Tower Wing, Guy’s Hospital, Great Maze Pond, London SE1 9RT, UK; 3 Department for Biochemistry and Molecular Biology (BMB), University of Southern Denmark, 5230 Odense, Denmark; ruppert@bmb.sdu.dk; 4 Department of Biochemistry-Biotechnology, School of Health Sciences, University of Thessaly, 41500 Larissa, Greece; dkouret@gmail.com

**Keywords:** ketosis, ketogenic diet, intermittent fasting, long-term fasting

## Abstract

Background: There is a growing consensus that fasting-induced ketosis has beneficial effects on human physiology. Despite these compelling benefits, fasting-induced ketosis raises concerns in some clinicians because it is often inappropriately compared with the pathologic uncontrolled ketone production in diabetic ketoacidosis. The determinants of the inter-individual differences in the intensity of ketosis during long-term fasting is unknown. Methods: We monitored daily variations in fasting ketonemia, as well as ketonuria, which is less invasive, in a large cohort of 1610 subjects, fasting between 4 and 21 days with the Buchinger Wilhelmi program, minimally supplemented with ~75–250 kcal (daily fruit juice, vegetable soup, and honey). Results: Ketonuria was detected in more than 95% of fasting subjects from day 4 onwards. Subjects consuming only soups, without fruit juice or honey, exhibited reduced caloric intake (72 kcal instead of 236 kcal) and carbohydrate intake (15.6 g instead of 56.5 g), leading to more intense ketonuria. Participants with high ketonuria were, in the majority, males, young, had a higher body weight, and had lower HDL-C and urea values. They had a larger decrease in blood glucose, glycated haemoglobin levels, body weight, and waist circumference. Furthermore, in the high-ketonuria group, a larger increase in blood uric acid concentration was observed. Conclusion: Our study showed that long-term fasting triggered ketosis, never reaching pathological levels, and that ketosis is influenced by age, gender, health, and the level of physical activity. Furthermore, it is modulated but not suppressed by minimal carbohydrate intake. Our study paves the way for better understanding how supplementation can modulate the therapeutic effects and tolerability of long-term fasting.

## 1. Introduction

Long-term fasting is one of the most potent non-pharmacological interventions for the prevention and management of chronic diseases [1,2]. In humans, it normalises lipid and glucose metabolism [3,4], has anti-inflammatory effects [5], reduces oxidative stress [6], and restores imbalances in the gut microbiota [7]. Studies in animals also showed the activation of autophagy, followed by cellular regeneration [8,9]. Despite these compelling benefits, fasting-induced ketosis raises concerns in some clinicians because it is often inappropriately compared with the pathologic uncontrolled ketone production in diabetic ketoacidosis [10]. Here, we address the safety of fasting-induced ketosis by evaluating the dynamics of ketosis and its modulation by age, gender, physiological status, physical activity, and minimal carbohydrate intake in a large cohort of 1610 individuals, as well as in another study of 32 individuals.

A hallmark of fasting is the metabolic switch from food-derived to endogenously sourced energy substrates. When food intake is interrupted, glucose levels and insulin levels drop to the lower levels of norms. The hormone glucagon is then released, signalling the liver to break down glycogen into glucose. A typical person has around 450 g of glycogen available, providing around 24 h of energy if this is the only source [10]. When glycogen stores are exhausted, lipolysis release fatty acids into the bloodstream as fuel for most of the body cells. In the liver, parts of fatty acids are transformed into energy-rich ketone bodies (acetoacetate, acetone, and β-hydroxybutyrate). Ketone bodies are water-soluble and can be transported to peripheral tissues (including the brain), where they are oxidised to allow for ATP production. Although proteins are not the primary source of energy during this process, a certain number of amino acids are mobilised for gluconeogenesis. This remains minimal, as protein-sparing mechanisms are activated when fatty-acid-derived water-soluble ketone bodies are produced.

Ketosis enables the brain to receive an adequate amount of fuel, and, at the same time, preserves lean mass [2,11]. Besides its function as an energy substrate, β-hydroxybutyrate is a signalling molecule that mediates adaptive responses to fasting [12,13]. β-hydroxybutyrate signals via G-protein-coupled receptors to modulate lipolysis and acts as an inhibitor of class I histone deacetylases [14]. Numerous health-promoting effects of fasting, such as reductions in oxidative stress [15] and inflammatory processes [16], were, at least in part, attributed to this production of ketone bodies in animal models. Other studies suggested that β-hydroxybutyrate downregulates sympathetic tone [17], induces vasodilation [18], and protect neurons from cellular damage by inducing the expression of brain-derived neurotrophic factor [19]. During fasting, mTOR is inactivated due to the absence of protein intake. In the particular case of muscles, the ketone body β-hydroxybutyrate inhibits the loss of muscle by activating mTORC1 [11]. This finding coincides with transcriptomic signatures elicited by β-hydroxybutyrate specifically in myocytes [20].

In the medical community, ketosis is often automatically linked to the pathologic uncontrolled condition of ketoacidosis [21]. Ketoacidosis is observed in patients with diabetes type 1 due to their incapacity to metabolise glucose, whereby the production of ketone bodies reaches extremely high levels, exceeding 20 mmol/L [10]. Ketoacidosis can also occur in type 2 diabetes [22], excess alcohol consumption, as a result of taking certain medications (e.g., SGLT2 inhibitors), or in end-stage starvation [10]. Uncontrolled ketosis leading to ketoacidosis is in sharp contrast with fasting ketosis, a physiological status where the production of ketone bodies is the key mechanism in order to spare proteins and thus be able to fast in the long-term [2,23]. Ketonemia can safely reach levels of approximately 4 mmol/L during fasting because evolution has equipped our cells with adaptative mechanisms to tolerate increased levels of ketone bodies and prevent the development of ketoacidosis. Insulin is a major regulator in this process [24]. The nuclear receptor peroxisome proliferator-activated receptor α (PPARα), enhanced during fasting, is the main regulator of this process, which, upon activation, transactivates genes encoding enzymes from the ketogenic pathway [25]. This feedback regulation prevents excessive and uncontrolled ketone production [24].

There are inter-individual differences in the intensity of ketosis during long-term fasting [26]. Their origin is largely unknown. We measured these inter-individual differences in the dynamics of ketosis in a large cohort of 1610 subjects and tested whether there is a relation between ketonuria and clinical, demographic, or dietary factors during long-term fasting. Since this study was conducted in humans, urine measurement of ketones was favoured because it is not invasive. This approach was validated by correlating ketonuria (i.e., ketone concentrations measured in urine) and ketonemia (i.e., ketone concentrations measured in blood) in a group of 32 patients. Last but not least, we evaluated if fasting effects were more pronounced in subjects with high ketonuria in comparison to subjects with low ketonuria. Altogether, our study reveals some factors influencing ketosis and its important role in the beneficial effects of long-term fasting.

## 2. Materials and Methods

### 2.1. Participants

Subjects were analysed in this observational study, as described in detail [3,27]. Included were subjects aged between 18 and 91 years who participated in a multidisciplinary fasting program at Buchinger Wilhelmi Clinic. A blood examination at the beginning of the stay was performed. Exclusion criteria were cachexia; anorexia nervosa; type 1 diabetes; advanced kidney, liver, or cerebrovascular insufficiency; dementia and other debilitating cognitive diseases; and pregnancy or lactation [28].

### 2.2. Fasting Program

All subjects underwent a medical examination prior to fasting. Fasting was undertaken according to the Buchinger Wilhelmi program under medical supervision, as previously described [3]. One day before fasting, the subjects consumed a 600 kcal vegetarian diet of either whole-grain rice and vegetables or fruits that was served in 3 meals. A laxative was administered in the morning of the first fasting day. During the fasting course, an enema was administered every second day. During fasting, 250 mL of organic fresh fruit juice and 250 mL of vegetable soup were delivered, as well as 20 g of honey. Some subjects preferred taking the soup two times, leading to a caloric intake of only 72 kcal/day. Subjects were instructed to drink 2–3 L/day of non-caloric water or herbal teas. The total calorie intake was 75–250 kcal/day. The fasting program included daily physical activity, alternating with rest in a setting that promoted calmness and mindfulness. On the last fasting day, ovo-lacto-vegetarian food was stepwise reintroduced, progressively increasing during four days from 800 to 1600 kcal/day.

### 2.3. Ketonemia and Ketonuria

For the measurement of ketonemia, samples were obtained by a prick into the fingertip with a lancet. Ketonemia levels were evaluated with the GlucoMen areo 2K (Berlin-Chemie AG, Berlin, Germany). Sodium nitroprusside tests allowed for a non-invasive detection of acetoacetate excretion in urine and a semi-quantitative evaluation [29]. Ketosis was evaluated with Ketostix strips (Bayer AG, Leverkusen, Germany). This imposes limitations. The nitroprusside test using Ketostix strips is known to be a reliable method to distinguish ketotic patients from nonketotic patients. However, only a few discrete quantitative values are provided (0 mg/dL, negative; 5 mg/dL, traces; 15 mg/dL, small; 40 mg/dL, moderate; 80 and 160 mg/dL, large). We considered ketonuria measures not as quantitative values but as categorical values representing ‘intensities’, like in the original study describing the reliability of Ketostix strips [30].

### 2.4. Clinical Data

Subjects documented the intake of fasting supplements in a diary. Physical and emotional well-being was evaluated on numeric rating scales from 0 (very bad) to 10 (excellent). The Well-being Index (WHO-5) was assessed [31]. Physical activity was measured with a visual analogue score (0–10). Body weight was documented daily on Seca 704 scales (Seca, Hamburg, Germany), while subjects were lightly dressed. Moreover, blood pressure was measured by nurses after a pause in the non-dominant arm while in a sitting position (boso Carat professional, BOSCH + SOHN GmbH u. Co. KG, Jungingen, Germany). Waist circumference was assessed before and at the end of fasting. Blood samples were collected before and at the end of fasting, as described previously [3]. A blood analysis was conducted according to international methods, as previously described [3]. The parameters measured included lipid profiles (total cholesterol, triglycerides, high-density lipoprotein cholesterol, low-density lipoprotein cholesterol), glycaemic indicators (blood glucose and glycated haemoglobin), blood count components (erythrocytes, haemoglobin, haematocrit, mean corpuscular haemoglobin, and thrombocytes), and thyroid-stimulating hormone. Coagulation parameters were also assessed (international normalized ratio, the Quick test, and partial thromboplastin time). Liver function tests measured serum glutamic oxaloacetic transaminase, serum glutamate pyruvate transaminase, serum gamma-glutamyl transferase, and alkaline phosphatase. Inflammatory biomarkers included C-reactive protein and erythrocyte sedimentation rate at 1 and 2 h. Renal function was evaluated by measuring uric acid, urea, and creatinine levels, and electrolyte levels were determined for sodium, potassium, calcium, and magnesium.

### 2.5. Statistics

The statistical analysis was performed in the R (v 4.0) open-source statistical environment [32]. Correlation analyses were performed using the Spearman’s rank correlation method in order to take the fact that ketonuria is not normally distributed into account. The calorie loss following ketone body production was calculated using 24 h urinary volumes collected daily during the study and the calorific value of ketone bodies determined in other studies [33]. The ketonemia increase rate was determined by fitting a robust regression model. Unsupervised hierarchical clustering of ketonuria computed from Euclidean distance was performed with Ward’s linkage algorithm using R Base package (v 4.0). The heatmap displaying this clustering was prepared using the heatmap.2 function from the ggplot package (v 3.5). The influence of food consumption was calculated using contingency tables, considering the consumption of honey and juice as a categorical variable. A mosaic plot showing an area-proportional visualization of observed frequencies was elaborated with R package vcd (Visualizing Categorical Data) (v 1.4.11). The *p*-values from the contingency tables were computed from a Chi-squared distribution. Random Forest classification was performed using R package Caret (version 6.0-84) [34]. Since the two classes were not balanced, down-sampling was performed prior to processing. Input variables were scaled and centred. The optimisation of mtry was performed with default parameters. Accuracy was estimated using repeated cross-validation (10-fold, repeated 10 times). The model was trained using 80% of the dataset, while the quality of this model was evaluated using predicted sample classification of the remaining 20% of the dataset. The quality control metrics were calculated using the confusionMatrix function from Caret. This function calculates the overall accuracy rate along a 95% confidence interval, with statistical significance of this accuracy evaluated with a one-side test comparing the experimental accuracy to the ‘no information rate’. Differences in the effects of fasting between the different clusters was computed as the estimated marginal means of linear models of mean change values for the two groups with low or high ketonuria (beta + standard error, SE) as a predictor of metabolic changes, with fasting duration and age as a covariate.

## 3. Results

**Figure 1:**
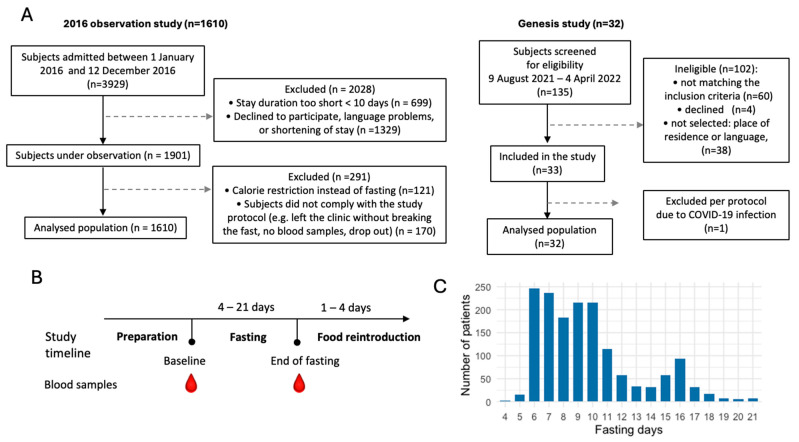
Flowchart of the study populations. (**A**). Subjects were part of two observational studies including 1610 and 32 subjects. (**B**). Study timeline. (**C**). Distribution of the fasting duration.

**Figure 2:**
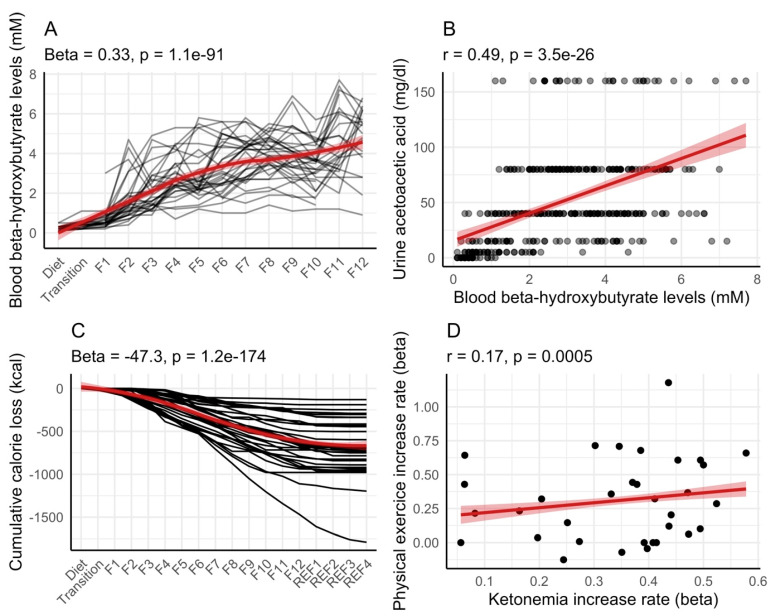
Correlation between ketonemia and ketonuria in 32 individuals during long-term fasting. This group of participants performed a 12-day fast (F1 up to F12) followed by 4 days of food reintroduction (REF1 to REF4). Ketonemia measured as the concentration in ß-hydroxybutyrate in blood (**A**) and ketonuria measured as the concentration in acetoacetate in urine were correlated (**B**). Cumulative calorie loss estimated from ketonuria and 24 h urine volume (**C**). An increased amount of physical exercise caused ketonemia to increase more rapidly (**D**). Red lines with shaded areas (95% confidence level interval) help in visualizing the general trends by fitting a smooth line (**A**,**C**), or a linear regression (**B**,**D**).

We evaluated the dynamics of ketosis during long-term fasting in 1610 individuals (Figure 1). Ketosis was monitored by the semi-quantitative measure of urine acetoacetate concentrations with sodium nitroprusside test strips, which is non-invasive. It was not clear if the results of ketonuria correlate well with ketonemia during long-term fasting. We thus compared these two methods in 32 subjects. Although measurement errors were larger for ketonuria because of the semi-quantitative nature of the method, these measures correlated well with the concentration in ß-hydroxybutyrate measured in blood (Figure 2A,B). The comparison between ketonuria and ketonemia in 32 subjects thus showed that the semi-quantitative measure of acetoacetate concentration in urine as a non-invasive, convenient procedure represents an accurate reflection of ketosis during long-term fasting.

In our cohort of 32 subjects, 24 h urine samples were collected. We estimated how many calories in the form of ketone bodies were lost in urine (Figure 2C). On average, participants lost 56.2 ± 39.4 kcal in the form of ketone bodies for each day of fasting (calculated from the 4th day of fasting when ketosis is well established). The cumulative loss of calorie during fasting was variable, ranging from 124 kcal to 1468 kcal.

We evaluated whether physical exercise during fasting could explain inter-individual differences in ketonemia. Interestingly, participants performing the most exercise during fasting had the fastest increase in ketonemia in this group of 32 persons (Figure 2D).

**Figure 3:**
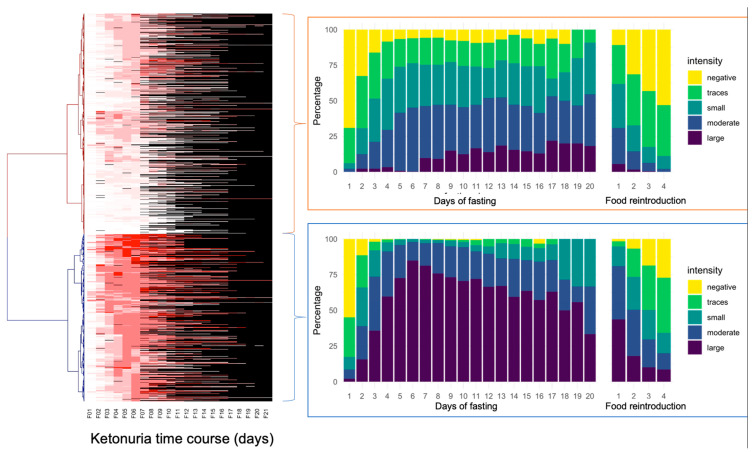
Differences in the intensity of ketonuria during 20 days of fasting in the cohort of 1610 individuals. The heatmap represents individual ketonuria values as a colour scale (not available missing values in black) during 20 days of fasting (F01 up to F21). Hierarchical clustering of Euclidian distances performed using the Ward function highlights two groups separating according to the time of onset of ketonuria.

**Figure 4:**
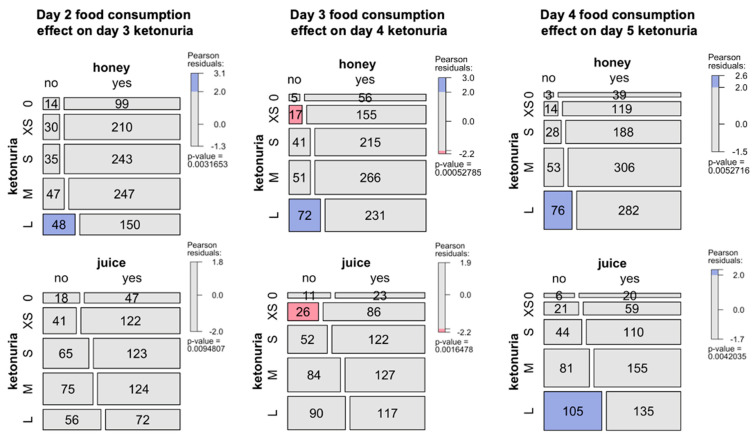
Influence of food consumption during fasting in the cohort of 1610 patients. Effects of the consumption of honey and juice during the second, third, or fourth day of fasting and its effects on next day ketonuria (0, none, XS, traces; S, small; M, medium; L, large). The mosaic plot shows an area-proportional visualization of observed frequencies. The *p*-value is computed from a Chi-squared distribution. Shadings for the contingency tables indicates the Pearson residuals, reflecting to which extent the observed values deviate from expected values.

The study of ketonuria during the first 20 days of long-term fasting in 1610 individuals showed that some individuals only excreted traces of ketones during fasting, while some rapidly excreted large concentrations of ketones after a few days (Table 1). Patients could be clustered as low ketonuric or high ketonuric (Figure 3). Since patients were asked to consume up to 75–250 kcal during their fast supplements, mainly providing carbohydrates (juice, clear soups, and honey), we hypothesised that differences in ketonuria could be due to this daily intake of carbohydrates. The composition of the single supplements is shown in Table 2. Soup could be chosen twice per day instead of juice at noon. We found that ketonuria was higher in individuals who did not consume honey and juices (Figure 4). By contrast, clear soup had no influence on ketonuria. We evaluated this relationship in more detail. A subgroup of 179 individuals without missing data described their supplement intake during the first 5 days. Classifying this caloric intake by quartiles confirmed the influence of supplement intake on ketonuria. The group representing the lowest quartile with 45 individuals, having an average daily caloric intake of 98 kcal, included 14 low-ketonuria and 31 high-ketonuria individuals. This corresponds to patients who did not consume honey or juice but only soup for lunch or dinner. By contrast, the group of the highest quartile with 44 individuals having an average daily caloric intake of 228 kcal included 25 low-ketonuria and 19 high-ketonuria individuals. We found 14 participants who reported having no ketones in their urine at any time during their stay.

**Figure 5:**
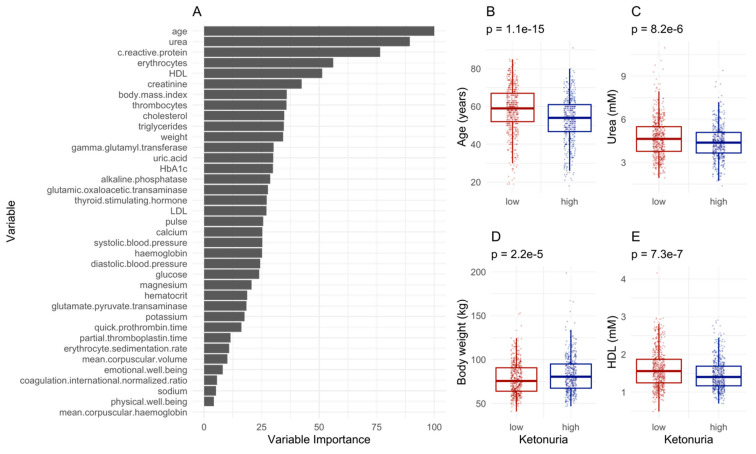
Machine learning predicts differences in ketonuria during long-term fasting. (**A**). The most important variables for sample classification. (**B**–**E**). Differences for the levels of the variables predicting the separation according to the time of onset of ketonuria. The *p*-value of a *t*-test is displayed.

We tested if other factors could influence ketonuria, such as demographic or blood biochemistry data. The best model classifying the patients as having a low ketonuria or a high ketonuria (accuracy = 0.63, 95% CI [0.58–0.69], *p*-value = 0.006) was driven by age differences, urea, weight, and erythrocytes, with HDL also contributing to the differences (Figure 5A). Males also had higher ketonuria scores. On average, patients with high ketonuria were younger (Figure 5B), had lower urea levels (Figure 5C) and lower HDL levels (Figure 5E), but higher body weight (Figure 5D). The inclusion of honey and juice intake data in the models improved the predictivity of the model (accuracy = 0.67, 95% CI [0.60–0.71], *p*-value = 0.0001). This suggested that both baseline differences in physiological and demographic parameters, as well as the ingestion of supplements are important in predicting ketonuria.

**Figure 6:**
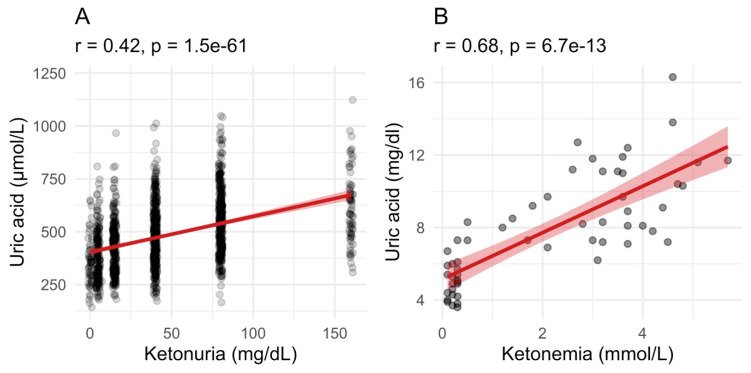
Uric acid levels correlate with ketonuria and ketonemia. (**A**). Uric acid levels during fasting correlated well with ketonuria in the group of 1610 patients between the start and the end of long-term fasting. (**B**). In the group of 32 patients where ketonemia was measured, the relationship between the retention of uric acid and the increase in blood ketone bodies level is even stronger. The red line with shaded area is a linear model with 95% confidence level interval.

We evaluated if fasting had different influences on blood parameters between the groups with high ketonuria or low ketonuria. The decrease in glucose and glycated haemoglobin (HbA1c) levels caused by fasting were less pronounced in the low-ketonuria group. In the group of patients with the lowest ketonuria, blood glucose levels decreased from 5.4 to 4.8 mmol/L. By contrast, in the highest ketonuria group, blood glucose levels decreased from 5.4 to 4.4 mmol/L (Table 3). This is not surprising since juice and honey provide 32.5 g and 16.2 g of carbohydrates, respectively. Similarly, changes in waist circumference and body weight were more pronounced in the high-ketonuria group. The metabolic parameter that was the most impacted was uric acid levels. While blood uric acid levels increased by 97 μmol/L in the low-ketonuria group, they increased by 206 μmol/L in the high-ketonuria group (Table 3). Changes in ketonuria were strongly correlated (r = 0.48, *p* = 2.2 × 10^−16^) with changes in uric acid levels (Figure 6). No differences between the high- and the low-ketonuria groups were measured for cholesterol (total, HDL, and LDL), triglyceride levels, well-being, or blood pressure.

## 4. Discussion

The therapeutic effects of long-term fasting are well documented [3]. Increases in ketonemia and the onset of ketonuria are a sign that the metabolic switch mainly from food glucose to endogenous energy substrates takes place. Assessing ketonuria instead of ketonemia is a simple non-invasive measurement that can be employed to determine the metabolic switch to the fasting mode during long-term fasting. Since in our clinical experience, we saw interindividual differences in ketonuria, we were interested in investigating the factors that could be responsible for these differences.

Our study with 32 patients showed that ketonemia steadily increased from around day 2 until the last day of the fast (12th day), where it reached safe average concentrations of around 4 mmol/L. Ketonemia levels were comparable with those in a recent study of 17 patients undergoing a 7.5 ± 2.9 day fast with the same similarly minimally supplemented diet [26]. In another study with water-only fasting, ketonemia became detectable after 21.1 h without exercise and 17.5 h with exercise [35]. This suggests that short fasting windows, typically lasting 12–16 h or less, would not trigger ketosis and that longer periods of fasting are more efficient to elevate the concentration of blood ketone bodies.

The establishment of ketosis could explain the decrease in hunger sensation that improves tolerability and compliance with fasting. In a prior study involving 1422 participants, we found that long-term fasting leads to a reduction in hunger levels compared to the hunger experienced on the day preceding the fast [3]. Several studies have correlated this diminishment of hunger with the increase in ketonemia, which directly suppress appetite [36]. Caloric restriction regimens that do not reach ketosis are thus probably more difficult to tolerate than long-term fasting, which triggers adaptative mechanisms, suppressing appetite.

Furthermore, whereas in fasting animals, ketonemia is routinely measured, in humans, non-invasive ketonuria measurement has more chances of enhancing compliance during long-term fasting. Having an objective visual signal that the body undergoes a series of hormonal and metabolic adaptations during fasting can empower individuals to navigate through the fasting period with a sense of control and safety. This is probably, at least in part, why fasting increases well-being [3] and self-efficacy [37] and has an empowering influence on a person’s lifestyle [38].

The measurement of ketone bodies in urine with the Ketostix strips was also not without its limitations. Compared to ketonemia, ketonuria reached a plateau around day 5. It is not clear if this was an artefact due to the limits of the Ketostix strips, which provide discrete quantitative values with a maximum of 160 mg/dL. On the other hand, the submaximal values also reached a plateau, which would suggest that, in this case, the Ketostix strips’ signal did not saturate. In our study, we estimated that approximately 60 kcal of ketones were lost every day in urine. Only 14 patients never reported ketonuria, and we hypothesised that either these persons encountered a problem with the use of the Ketostix strips, or their ketone bodies were completely metabolised for energy production, or else their ketone bodies were completely reabsorbed by the kidneys.

How come we excrete energy-rich compounds like ketones during fasting? The excretion of ketone bodies in urine during fasting seems contradictory to the body’s energy conservation efforts. The kidneys are capable of reabsorbing part of these ketone bodies, but when a threshold is surpassed, they are eliminated [29]. During fasting, the rate of ketone body production may exceed the rate of utilization, leading to an excess that is excreted. During water-only fasting in obese individuals, ketone bodies’ rate of production of 2.5 mmol/min was only slightly above the maximal rate of disappearance (metabolic uptake) by tissues (2.3 mmol/min). Studies estimated that only 2–5% of the ketone bodies produced are excreted in urine [39]; hence, in our cohorts, the presence of ketonuria per se is in line with the established literature. Although this energy loss is not relevant for 5 to 20 days—or sometimes more, like in the case of long-term fasting—it could be speculated that it can become relevant in the case of a ketogenic diet, which is maintained for a longer period of time and for which ketonuria is also documented [40]. In a cohort of Irish adults with severe obesity at a specialist bariatric clinic, weight loss at eight weeks was proportional to increases in fasting beta-hydroxybutyrate after two and eight weeks with a low-energy, milk-based meal replacement [41]. Calorie loss could contribute to the total weight loss.

The fasting regimen that was used here was minimally supplemented with beverages that, according to the individual’s choice, provides 75–250 kcal. Subjects who consumed honey or/and juices (16–33 g carbohydrates) presented less intense ketonuria than subjects who did not consume these supplements. This finding is in line with previous studies investigating the effect of carbohydrate intake on ketonuria in long-term water-only fasting [42]. It seems logical that carbohydrate ingestion slowed down the production rate of total ketones via known suppressive effects of glucose intake and insulin signalling on ketogenesis. However, one study observed changes in ketonuria in the absence of changes in ketonemia, circulating glucose, or insulin with 7.5 g carbohydrate ingestion [42]. This suggests that the modulation of ketonuria by carbohydrate intake depends on the total amount of carbohydrates ingested and can involve hitherto unknown signals that bypass known metabolic processes to acutely regulate ketonuria. Since fuel switches during fasting occur, the decrease in glucose levels was more prominent in individuals with higher ketonuria, while cholesterol and triglyceride levels remained relatively stable regardless of the extent of ketosis. Future studies can include continuous glucose and ketone monitoring to better study the effects of fasting and the large inter-individual variability in postprandial responses [43].

In our study, we could confirm a strong correlation between ketonuria and uricemia. It is already known from previous research that as ketone levels in the blood increase, the excretion of uric acid in urine decreases. This happens because ketones and uric acid compete for the same transport mechanisms in the kidneys [44]. Clinically, high uric acid levels are often seen as a problem because they can lead to gout attacks. By contrast, we observed only one case of a gout attack in a patient treated for hyperuricemia in a large cohort of 1422 individuals fasting for up to 21 days, despite high elevations of uric acid (from 338 to 495 µmol/L). Importantly, uric acid acts as a potent antioxidant [45]. In line with our previous findings, we observed that fasting for an extended period led to an increase in the body’s total antioxidant capacity and a decrease in lipid peroxidation [6]. Additionally, the ketone β-hydroxybutyrate has been shown to suppress the activation of the NLRP3 inflammasome [16], thereby reducing the inflammatory response to urate crystals. By contrast, rats that received allopurinol to lower uric acid production experienced reduced physical performance and increased oxidative stress [46].

Consequently, the increase in uric acid levels was also more important in the group with high ketonuria. It could be hypothesized that patients with the highest metabolic health benefited the most from fasting because their metabolic abilities were higher. For parameters like urea, the greater decrease observed in the low-ketonuria group corresponds to their higher baseline levels. This suggests that the greater tendency to normalisation in these parameters could be due to the fact that individuals with higher baseline values—possibly more pathological—may have lower metabolic flexibility, leading to lower ketosis. This is supported by a study of metabolic function in 16,523 Korean subjects, indicating that the odds of having obesity was increased in the non-ketonuria group compared to the ketonuria group [47]. The authors interpreted this finding as an indication that higher ketonuria is indicative of metabolic superiority, and they speculated that individuals with high ketonuria could have a higher fat oxidation ability [47]. Given the known antioxidant capacities of uric acid, it could also be postulated that patients with high ketonuria have a better antioxidant status than patients with low ketonuria via the increase in uric acid, which could point to an evolutionary adaptation by which the loss of ketone bodies in urine is also a way to maintain antioxidant capacity in the fasting body receiving no exogenous antioxidants from food.

## 5. Conclusions

In conclusion, our study showed that long-term fasting triggered ketosis, never reaching unhealthy physiological levels, that ketosis is influenced by age, gender, physiological status, and the level of physical activity, and that it is modulated but not suppressed by minimal carbohydrate intake. Our study also corroborates the need to establish norm values for persons in a fasting mode, which often differ from the norm values of persons in an eating mode. This would avoid unjustified safety concerns caused by inappropriate comparisons to the metabolism during the eating mode. Overall, our study lays a foundation suggesting that a person’s characteristics can be used to predict the outcome of long-term fasting, paving the way to the establishment of personalised long-term fasting strategies.

## Figures and Tables

**Table 1:** Differences in the intensity of ketonuria during 20 days of fasting. Ketonuria values were reported from the measurement with Ketostix (0 mg/dL, negative; 5 mg/dL, traces; 15 mg/dL, small; 40 mg/dL, moderate; 80 and 160 mg/dL, large).

**Intensity**	**F1**	**F2**	**F3**	**F4**	**F5**	**F6**	**F7**	**F8**	**F9**	**F10**
negative	62%	24%	10%	5%	4%	4%	3%	3%	4%	5%
traces	26%	30%	21%	15%	12%	10%	11%	11%	9%	11%
small	6%	22%	25%	23%	20%	19%	18%	17%	19%	18%
moderate	4%	16%	26%	29%	32%	30%	27%	30%	28%	29%
large	1%	8%	17%	27%	32%	37%	40%	38%	39%	37%
**Intensity**	**F11**	**F12**	**F13**	**F14**	**F15**	**F16**	**F17**	**F18**	**F19**	**F20**
negative	5%	5%	4%	2%	3%	5%	2%	4%	2%	0%
traces	11%	13%	10%	13%	14%	11%	15%	11%	13%	8%
small	17%	14%	18%	20%	18%	22%	17%	26%	25%	28%
moderate	24%	30%	28%	30%	28%	28%	26%	28%	27%	28%
large	42%	38%	40%	35%	36%	34%	40%	32%	33%	36%

**Table 2:** Nutrient composition of the fasting supplements.

	Amount	Fat (g)	Protein (G)	Carbo-Hydrate (G)	Fibres (g)	Calories (kcal)
juice	250 mL	0.1	0.8	32.5	0.3	133.5
broth	250 mL	0.1	1.0	7.8	1.0	36
honey	20 g	-	-	16.2	-	64.8
total		0.1	1.8	56.5	1.3	234.4

**Table 3:** Metabolic effects of long-term fasting are different between patients with low or high ketonuria. Estimated marginal means of the linear models for the difference in mean change value for the two groups with low or high ketonuria (beta + standard error, SE) as a predictor of metabolic changes, with fasting duration and age as a covariate. *p*-values are provided (bold characters, *p* < 0.05).

Parameter	Low Ketonuria	High Ketonuria	*p*-Value
waist circumference decrease (cm)	−4.98 ± 0.19	−5.76 ± 0.20	**5.6 × 10^−3^**
weight decrease (kg)	−3.77 ± 0.06	−4.44 ± 0.07	**2.6 × 10^−12^**
systolic blood pressure decrease (mmHg)	−8.09 ± 0.658	−6.43 ± 0.711	9.3 × 10^−2^
diastolic blood pressure decrease (mmHg)	−4.26 ± 0.41	−3.68 ± 0.44	3.4 × 10^−1^
HDL decrease (mmol/L)	−0.235 ± 0.01	−0.203 ± 0.01	5.9 × 10^−2^
LDL decrease (mmol/L)	−0.289 ± 0.033	−0.363 ± 0.035	1.3 × 10^−1^
triglyceride decrease (mmol/L)	−0.437 ± 0.03	−0.391 ± 0.03	3.4 × 10^−1^
total cholesterol decrease (mmol/L)	−0.631 ± 0.03	−0.663 ± 0.03	5.0 × 10^−1^
uric acid increase (μmol/L)	+100 ± 4.52	+200 ± 4.88	**1.1 × 10^−44^**
urea decrease (mmol/L)	−1.74 ± 0.05	−1.29 ± 0.06	**1.6 × 10^−8^**
glucose decrease (mmol/L)	−0.539 ± 0.06	−0.970 ±0.06	**1.1 × 10^−6^**
hba1c (mmol/mol)	−0.112 ± 0.01	−0.160 ± 0.01	**1.7 × 10^−3^**
well-being index (WHO-5) increase	+17.8 ± 0.979	+20.3 ± 1.024	7.7 × 10^−2^
emotional well-being increase	+1.60 ± 0.09	+1.77 ± 0.10	1.9 × 10^−1^
physical well-being increase	+1.80 ± 0.10	+2.02 ± 0.10	1.2 × 10^−1^

## Data Availability

Data are contained within the article or Supplementary Materials.

## Supplementary Materials

The following supporting information can be downloaded at: https://www.mdpi.com/article/10.3390/nu16121849/s1. This file contains raw data for the measurements performed in all participants.
